# The pharmacological properties of 3-arm or 4-arm DOTA constructs for conjugation to α-melanocyte-stimulating hormone analogues for melanoma imaging

**DOI:** 10.1371/journal.pone.0213397

**Published:** 2019-03-22

**Authors:** Masato Kobayashi, Toshitaka Kato, Kohshin Washiyama, Masaaki Ihara, Asuka Mizutani, Kodai Nishi, Leo G. Flores, Ryuichi Nishii, Keiichi Kawai

**Affiliations:** 1 Wellness Promotion Science Center, Institute of Medical, Pharmaceutical and Health Sciences, Kanazawa University, Kanazawa, Japan; 2 Department of Health Sciences, Institute of Medical, Pharmaceutical and Health Sciences, Kanazawa University, Kanazawa, Japan; 3 Advanced Clinical Research Center, Fukushima Global Medical Science Center, Fukushima Medical University, Fukushima, Japan; 4 Department of Radioisotope Medicine, Atomic Bomb Disease Institute, Nagasaki University, Nagasaki, Japan; 5 Department of Pediatrics, MD Anderson Cancer Center, Houston, Texas, United States of America; 6 Molecular Imaging Center, National Institute of Radiological Sciences, Chiba, Japan; 7 Biomedical Imaging Research Center, University of Fukui, Fukui, Japan; Northwestern University Feinberg School of Medicine, UNITED STATES

## Abstract

**Background:**

Although a 3-arm DOTA construct, which has three carboxylic acids, h has been applied for conjugation to many peptides, we investigated if a 4-arm DOTA construct conjugated to peptides improves chemical properties for melanoma imaging of the melanocortin 1 receptor compared to 3-arm DOTA-conjugated peptides.

**Methods:**

Specific activities, radiolabeling efficiencies, and partition coefficients were evaluated using ^111^In-labeled 3-arm and 4-arm DOTA-α-melanocyte-stimulating hormone (MSH). For assessment of MC1-R affinity and accumulation in tumor cells in vitro, B16-F1 melanoma and/or 4T1 breast cancer cells were incubated with ^111^In-labeled 3-arm and 4-arm DOTA-α-MSH with and without α-MSH as a substrate. The stability was evaluated using mouse liver homogenates and plasma. Biological distribution and whole-body single photon emission computed tomography imaging of ^111^In-labeled 3-arm and 4-arm DOTA-α-MSH were obtained using B16-F1 melanoma-bearing mice.

**Results:**

Specific activities and radiolabeling efficiencies of both radiotracers were about 1.2 MBq/nM and 90–95%, respectively. The partition coefficients were −0.28 ± 0.03 for ^111^In-labeled 3-arm DOTA-α-MSH and −0.13 ± 0.04 for ^111^In-labeled 4-arm DOTA-α-MSH. Although accumulation was significantly inhibited by α-MSH in B16-F1 cells, the inhibition rate of ^111^In-labeled 4-arm DOTA-α-MSH was lower than that of ^111^In-labeled 3-arm DOTA-α-MSH. ^111^In-labeled 4-arm DOTA-α-MSH was taken up early into B16-F1 cells and showed higher accumulation than ^111^In-labeled 3-arm DOTA-α-MSH after 10 min of incubation. Although these stabilities were relatively high, the stability of ^111^In-labeled 4-arm DOTA-α-MSH was higher than that of ^111^In-labeled 3-arm DOTA-α-MSH. Regarding biological distribution, ^111^In-labeled 4-arm DOTA-α-MSH showed significantly lower average renal accumulation (1.38-fold) and significantly higher average melanoma accumulation (1.32-fold) than ^111^In-labeled 3-arm DOTA-α-MSH at all acquisition times. ^111^In-labeled 4-arm DOTA-α-MSH showed significantly higher melanoma-to-kidney, melanoma-to-blood, and melanoma-to-muscle ratios than ^111^In-labeled 3-arm DOTA-α-MSH.

**Conclusions:**

The 4-arm DOTA construct has better chemical properties for peptide radiotracers than the 3-arm DOTA construct.

## Introduction

The incidence rate of malignant melanoma has been steadily increasing over the past 40 years. The 5-year survival rate with stage IV metastatic melanoma is currently less than 20% because few effective treatments have been established [1,2]. Because survival is associated with an earlier stage at detection and treatment, specific and highly detectable imaging of melanoma tumors is strongly desired.

The melanocortin 1 receptor (MC1-R) is one of the most targeted melanoma antigens and belongs to the melanocortin family of G protein-coupled receptors, which consists of five receptor subtypes, MC1-R to MC5-R. MC1-R is expressed in nearly all primary and metastatic melanomas [3] and 95% of uveal melanomas [4]. MC1-R is an attractive receptor for molecular-targeted imaging and radionuclide therapy of melanoma. α-melanocyte-stimulating hormone (α-MSH), a tridecapeptide, is an endogenous ligand for the melanocortin family of receptors, with subnanomolar binding affinity to MC1-R [5].

The native α-MSH peptide hormone (Ac-Ser^1^-Tyr^2^-Ser^3^-Met^4^-Glu^5^-His^6^-Phe^7^-Arg^8^-Trp^9^-Gly^10^-Lys^11^-Pro^12^-Val^13^-NH_2_) is proteolytically processed from proopiomelanocortin and is primarily responsible for regulation of skin pigmentation [6, 7]. Alpha-MSH peptides bind the MC1-R selectively with nanomolar to subnanomolar affinities [8, 9]. Although the native α-MSH peptide has been directly radiolabeled with radioiodine etc., it shows low specific activity, low MC1-R affinity [10], and poor stability [11]. The addition of non-natural amino acids yielded α-MSH analogues with greater affinity and stability [11].

**Fig 1:**
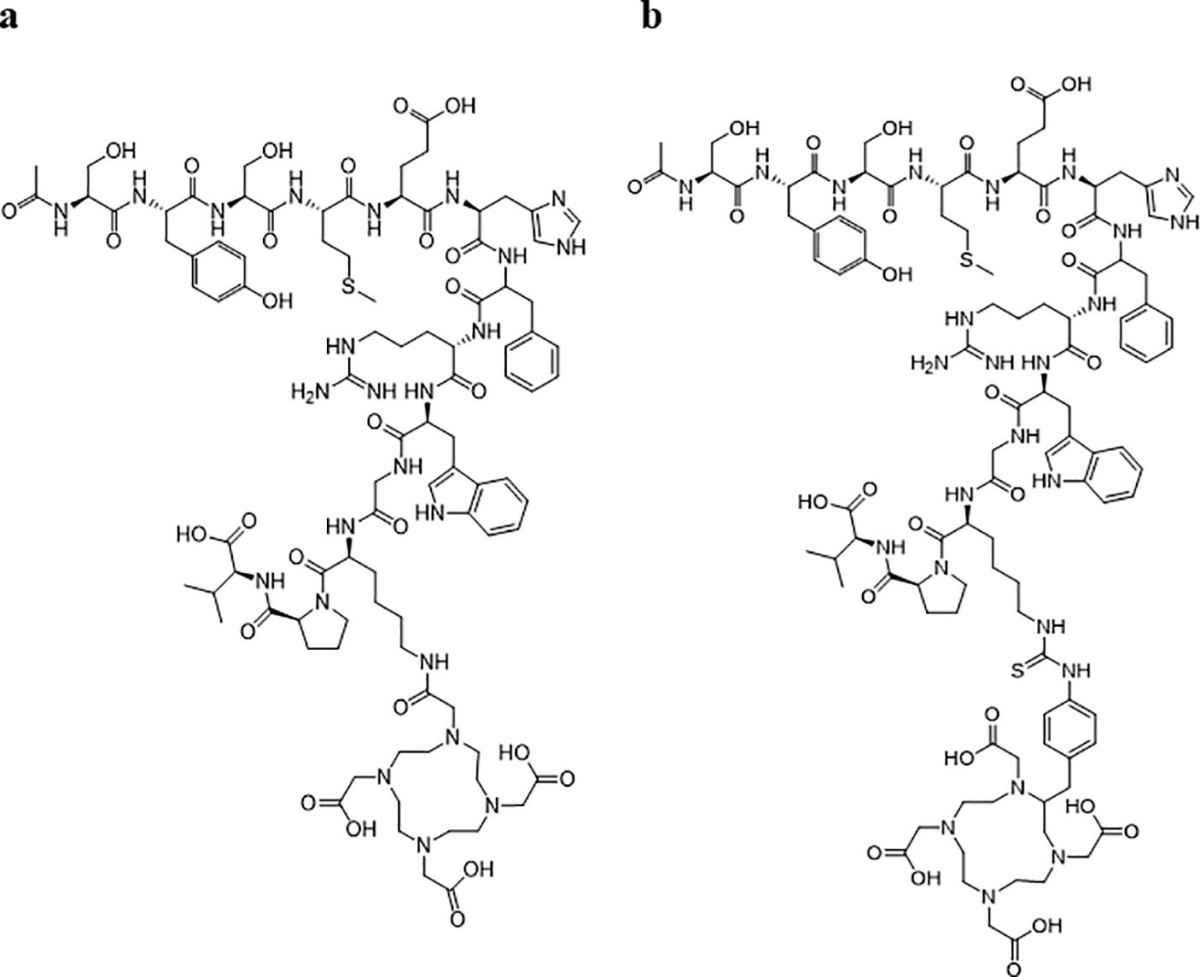
Structures of the 3-arm DOTA construct (a) and 4-arm DOTA construct (b). https://doi.org/10.6084/m9.figshare.7697861.

Radionuclide-labeled 1,4,7,10-tetraazacyclododecane-1,4,7,10-tetraacetic acid (DOTA)-chelate has been conjugated to many peptides and provides better specific activity, MC1-R affinity, and stability for melanoma imaging of MC1-R [12]. Although the 3-arm DOTA construct, which has three carboxylic acids, has been applied for conjugation to many peptides, the 4-arm DOTA construct, which has four carboxylic acids, improves radiochemical yield, specific activity, and stability of therapeutically active conjugates to antibodies for radioimmunotherapy of cancer [13]. In this study, we investigated if the 4-arm DOTA construct conjugated to peptides improves the chemical properties for melanoma imaging of MC1-R compared to 3-arm DOTA-conjugated peptides. Numerous α-MSH conjugated peptide analogues have been developed with high affinities and specificities for α-MSH receptors [14]. Native α-MSH, as an example of a peptide, was selected to evaluate improvement of chemical properties using the 4-arm DOTA construct because 3-arm DOTA-conjugated native α-MSH (3-arm DOTA-α-MSH, Fig 1A) yielded relatively low MC1-R affinity and stability compared to other 3-arm DOTA-conjugated peptides [14].

## Materials and methods

### Reagents and radionuclides

α-MSH free acid was purchased from Abcam (Cambridge, UK). The chelating agent DOTA and *N*-hydroxysuccinimidyl (NHS)-ester and *S*-2-(4-Isothiocyanatobenzyl)-1,4,7,10-tetraazacyclododecane tetraacetic acid (p-SCN-Bn-DOTA) were obtained from Macrocyclics, Inc. (Plano, TX, USA). ^111^InCl_3_ was purchased from FUJIFILM RI Pharma Co., Ltd. (Chiba, Japan).

### Synthesis and purification of 3-arm and 4-arm DOTA constructs

The conjugation procedure was performed using the methods of Maguire and Greg et al., with some modifications [13,15]. Briefly, to form the 3-arm DOTA-α-MSH, α-MSH free acid (1 eq) was dissolved in purified water (10 mg/mL solution) and conjugated to DOTA-NHS-ester (40 eq), which was dissolved in N,N-dimethylformamide (40 mg/mL solution), using 0.1 M NaHCO_3_ buffer (pH 7–8) for 6 hours at room temperature.

For 4-arm DOTA-conjugated native α-MSH (4-arm DOTA-α-MSH, Fig 1B), p-SCN-Bn-DOTA (10 eq) was dissolved in *N*,*N*-dimethylformamide (40 mg/mL solution). α-MSH free acid (1 eq, 10 μg/mL purified water solution) was conjugated to p-SCN-Bn-DOTA using 0.1 M NaHCO_3_ buffer (pH 7–8) for 24 hours at room temperature.

These resulting products were purified using high performance liquid chromatography (HPLC, Hitachi, Ibaraki, Japan) using a 5C18-AR-II column (Cosmosil, Nakalai Tesque, Kyoto, Japan), a combination gamma counter, and 210-nm ultraviolet light. For the 3-arm DOTA construct, the initial solvent was 0.05% trifluoroacetic acid in water (pH 2.2): acetonitrile at 78:22 after injection, and was gradually changed to 74:26 after 20 min as the eluent at a flow rate of 1.0 mL/min. For the 4-arm DOTA construct, the initial solvent was 0.05% trifluoroacetic acid in water (pH 2.2): acetonitrile at 78:22 after injection, and was gradually changed to 75:25 after 40 min as the eluent at a flow rate of 1.0 mL/min.

### Radiolabeling, purification, and partition coefficients

The 3-arm and 4-arm DOTA-α-MSH were labeled by the addition of ^111^InCl_3_ in 1 M CH_3_COONH_4_ buffer (pH 5.5, 100 mL) at 70°C for 5 min [16,17]. ^111^In-labeled 3-arm and 4-arm DOTA-α-MSH were purified by reversed phase-HPLC. For 3-arm DOTA-α-MSH, the initial solvent was 0.01% trifluoroacetic acid in water (pH 2.5): acetonitrile at 76:24, and was gradually changed to 75:25 after 20 min as the eluent at a flow rate of 1.0 mL/min and UV detector (895 V and 210 nm). For 4-arm DOTA-α-MSH, the solvent was 0.01% trifluoroacetic acid in water (pH 2.0): acetonitrile at 73:27.

The partition coefficients of ^111^In-labeled 3-arm and 4-arm DOTA-α-MSH were measured using 2.0 mL n-octanol as the organic phase and 2.0 mL 0.l M phosphate buffer as the aqueous phase (pH 7.4 for plasma) [18]. N-octanol and the buffer were pre-mixed twice using a mechanical mixer for 1 min at room temperature. Then, 20 υL radioactive sample were added and mixed twice using a mechanical mixer for 1 min at room temperature. The radioactivity of 200 uL of each phase was measured after centrifugation. Calculation of log (n-octanol/0.l M phosphate buffer) was performed.

### Tumor cells

Mouse skin melanoma B16-F1 cells with high MC1-R expression and mouse breast cancer 4T1 cells with low MC1-R expression were obtained from American Type Culture Collection. B16-F1 cells and 4T1 cells were cultured in Dulbecco’s Modified Eagle’s Medium (Wako, Osaka, Japan) and Roswell Park Memorial Institute-1640 (Wako), respectively, supplemented with 10% fetal bovine serum (Dainippon Sumitomo, Osaka, Japan) at 37°C in a 5% CO_2_ incubator.

### In vitro assays with B16-F1 and 4T1 cells

In vitro assays were performed using our methods, with some modifications [19]. Briefly, for the inhibition study, B16-F1 and 4T1 cells were seeded into 24-well cell culture multiwell plates at a density of 5×10^5^ cells/well. Assays were conducted 24 hours after seeding. B16-F1 and 4T1 cells were pre-incubated with each type of medium including 1.0 υM α-MSH for 180 min at 37°C. B16-F1 and 4T1 cells were incubated for 180 min with ^111^In-labeled 3-arm or 4-arm DOTA-α-MSH.

For the accumulation study with ^111^In-labeled 3-arm and 4-arm DOTA-α-MSH, each well was incubated with each type of medium for 10 min at 37°C. Then, 20 kBq/well ^111^In-labeled 3-arm or 4-arm DOTA-α-MSH was added and incubated for 10, 30, 60, 120, and 180 min at 37°C as the control condition. At the end of the incubation in the inhibition and accumulation studies, each well was rapidly washed twice with 500 μL 0.5 M HEPES buffer. Cells were then solubilized in 500 μL 0.1 M NaOH. The radioactivity that accumulated in B16-F1 and 4T1 cells was measured with a gamma counter (ARC-380; Hitachi-Aloka Medical, Tokyo, Japan). B16-F1 and 4T1 cells were detached with trypsin, and the protein in the cells was measured using a protein assay. All experimental conditions were examined with quadruplicate assays.

### Stability analysis of ^111^In-labeled 3-arm and 4-arm DOTA-α-MSH in mice

All animal studies were conducted following approval by the Animal Care Committee of Kanazawa University (AP-122339). Fasted B16-F1-bearing C57BL6 male mice (5 weeks old) were administered ^111^In-labeled 3-arm or 4-arm DOTA-α-MSH (3.7 MBq/mouse) via the tail vein. At 10, 30, 60, 120, and 180 min after injection, mice (n = 3 per time point) were euthanized with isoflurane, and blood and liver were collected and analyzed with thin layer chromatography (TLC). Briefly, 300 uL blood in a heparinized tube was centrifuged at 18,000 **×**g at 4°C for 5 min. A total of 30 uL perchloric acid was added to the supernatant, which was centrifuged again, after which the final supernatant was spotted onto the TLC plate as plasma. Krebs-Ringer phosphate buffer (pH 7.4) was added to the liver samples, followed by homogenization with an ultrasonic homogenizer (SONIFIER250, Branson, MO, USA). Then, ethanol was added to the homogenate to remove proteins, and the sample was centrifuged at 18,000 **×**g at 4°C for 5 min. The final supernatant was spotted onto the reverse-phase TLC plate, and the TLC spots were developed using 50 mM EDTA (pH 4–5) solvents. After development and complete drying, the TLC plates were cut into 21 fractions, and the radioactivity associated with each fraction was quantified using a γ-ray counter. The fraction ratios of ^111^In^3+^, ^111^In-labeled 3-arm or 4-arm DOTA-α-MSH, and all metabolites were calculated by dividing the radioactive counts for each fraction by the total radioactivity counts. A stability study showed that the fraction of ^111^In-labeled 3-arm or 4-arm DOTA-α-MSH remained the same at all injection times.

### Biological distribution and whole-body single photon emission computed tomography (SPECT) imaging of ^111^In-labeled 3-arm and 4-arm DOTA-α-MSH in B16-F1-bearing mice

C57BL6 male mice (5 weeks old) were transplanted with B16-F1 cells (5 × 10^5^ cells/100 μL) into the thigh of the mice [19]. The tumors appeared and gradually increased in size after about 10 days. The mice were housed and continuously monitored each day for 2 weeks in a 12-hour light/12-hour dark cycle with free access to food and water. The tumor size reached 8.7 ± 2.8 mm at 2 weeks after transplant of cells. Two mice with tumor sizes of 13 and 15 mm were excluded from our experiments. Mice with a tumor size <10 mm were fasted with no food overnight with water supplied ad libitum before experiments.

For biological distribution studies, B16-F1-bearing mice were administered ^111^In-labeled 3-arm or 4-arm DOTA-α-MSH via the tail vein (37 kBq/mouse). At 10, 30, 60, 120, and 180 min after injection, the mice were euthanized under isoflurane (n = 3 per time point), and the following tissues were collected: brain, lung, heart, stomach, liver, small intestine, large intestine, kidney, blood, muscle, and B16-F1 melanoma. Tissues were weighed, and radioactivity was quantified using an automated γ-ray counter to calculate the percent injected dose (%ID) or percent injected dose per gram of tissue (%ID/g).

For SPECT imaging, ^111^In-labeled 3-arm or 4-arm DOTA-α-MSH was injected into the tail vein of four B16-F1-bearing mice in total (37 MBq/mouse). For SPECT imaging, acquisition was started at 10 min after injection and continued every 10 min for 180 min. The data were reconstructed using the ordered subset expectation maximization method with 16 subsets and six iterations including no scatter and attenuation correction. The voxel size was set to 0.8×0.8×0.8 mm. Post-reconstruction smoothing filtering was applied using a 1.0-mm Gaussian filter. Image displays were obtained using medical image data analysis software, AMIDE (ver. 1.04). The coronal images were displayed as maximum intensity projections. In these images, three to five regions of interest were placed over the kidney, muscle, and B16-F1 melanoma in which accumulation could be seen, and the time activity curve of each was obtained as the %ID/g.

### Statistical analysis

Data are presented as the mean and standard deviation (SD). P values were calculated using a two-tailed Student’s *t*-test. Results were considered to be significant at *P* < 0.01.

## Results

### Specific activity, radiolabeling efficiency, and partition coefficients of ^111^In-labeled 3-arm or 4-arm DOTA-α-MSH

Specific activities of both ^111^In-labeled 3-arm and 4-arm DOTA-α-MSH were about 1.2 MBq/nM. With HPLC analysis, the retention times were 2.5–3.0 min for ^111^In^3+^, 14–17 min for ^111^In-labeled 3-arm DOTA-α-MSH, and 20–22 min for ^111^In-labeled 4-arm DOTA-α-MSH. Radiolabeling efficiencies of both ^111^In-labeled 3-arm and 4-arm DOTA-α-MSH were 90–95%. Non-carrier-added radiotracers for both compounds were obtained with a radio-chemical purity of >95% after purification. For TLC analysis of partition coefficients, the R_f_ values of ^111^In-labeled 3-arm DOTA-α-MSH, 4-arm DOTA-α-MSH, and ^111^In^3+^ were 0.35–0.45, 0.40–0.50, and 0.00, respectively. Partition coefficients were −0.28 ± 0.03 for ^111^In-labeled 3-arm DOTA-α-MSH and −0.13 ± 0.04 for ^111^In-labeled 4-arm DOTA-α-MSH.

### In vitro assays with B16-F1 and 4T1 cells

**Fig 2:**
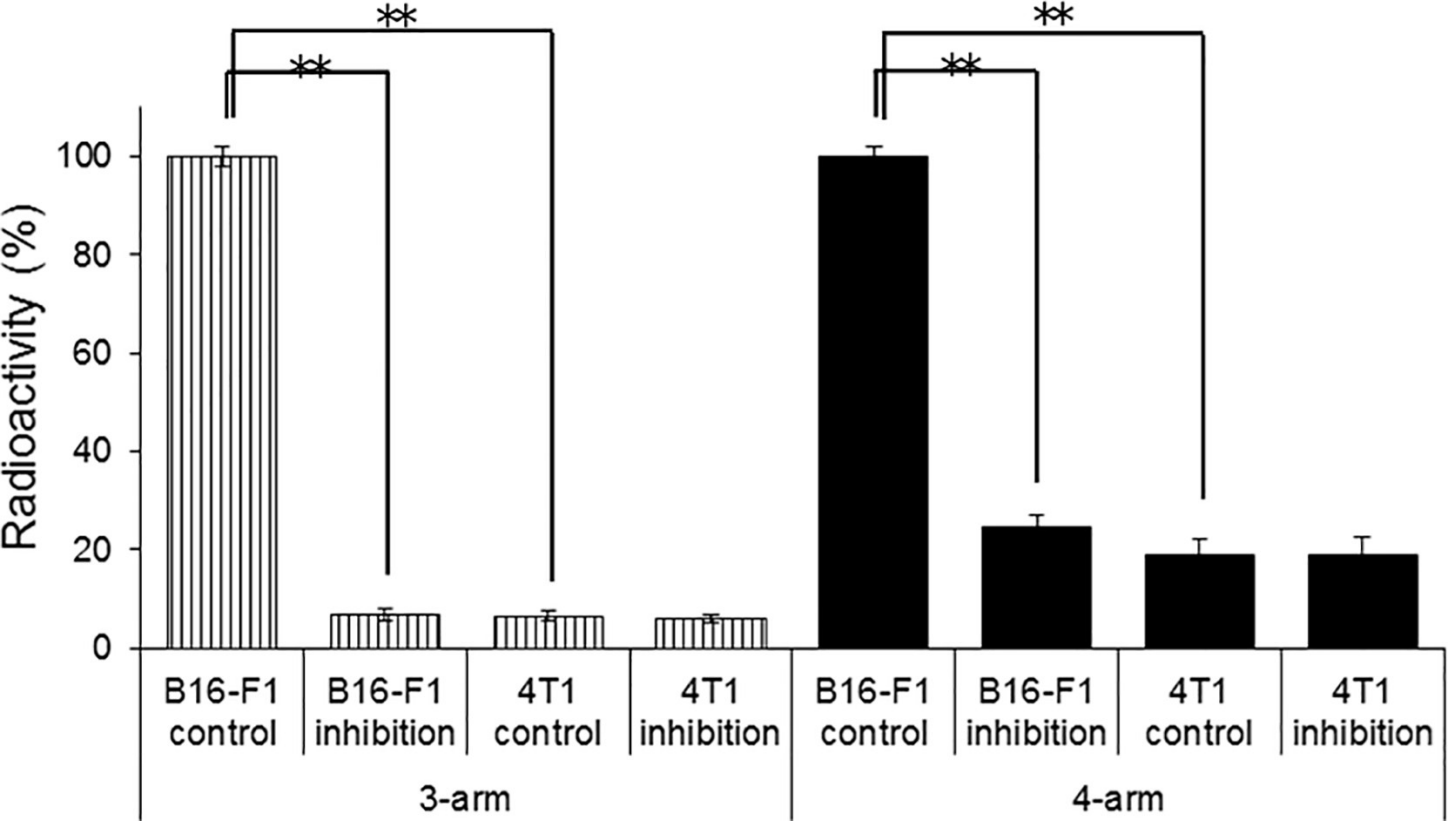
In vitro assays of ^111^In-labeled 3-arm DOTA-α-MSH and ^111^In-labeled 4-arm DOTA-α-MSH in B16-F1 melanoma cells and 4T1 breast cancer cells after 180 min of incubation in the presence or absence (control) of α-MSH. The accumulation of both radiotracers in cells was significantly inhibited by α-MSH. In 4T1 cells with low MC1-R expression, no significant accumulation and no inhibition effect were observed for either radiotracer. **P < 0.01 vs. control. https://doi.org/10.6084/m9.figshare.7697882.

Fig 2 shows the studies without and with the substrate of 1.0 μM α-MSH and ^111^In-labeled 3-arm and 4-arm DOTA-α-MSH in B16-F1 melanoma cells with high MC1-R expression and 4T1 breast cancer cells with low MC1-R expression measured with real-time qPCR. Accumulation of both ^111^In-labeled 3-arm and 4-arm DOTA-α-MSH was significantly inhibited by α-MSH in B16-F1 cells, but the inhibition rate of ^111^In-labeled 4-arm DOTA-α-MSH was lower than that of ^111^In-labeled 3-arm DOTA-α-MSH. In 4T1 cells, we found no significant accumulation of either radiotracer.

**Fig 3:**
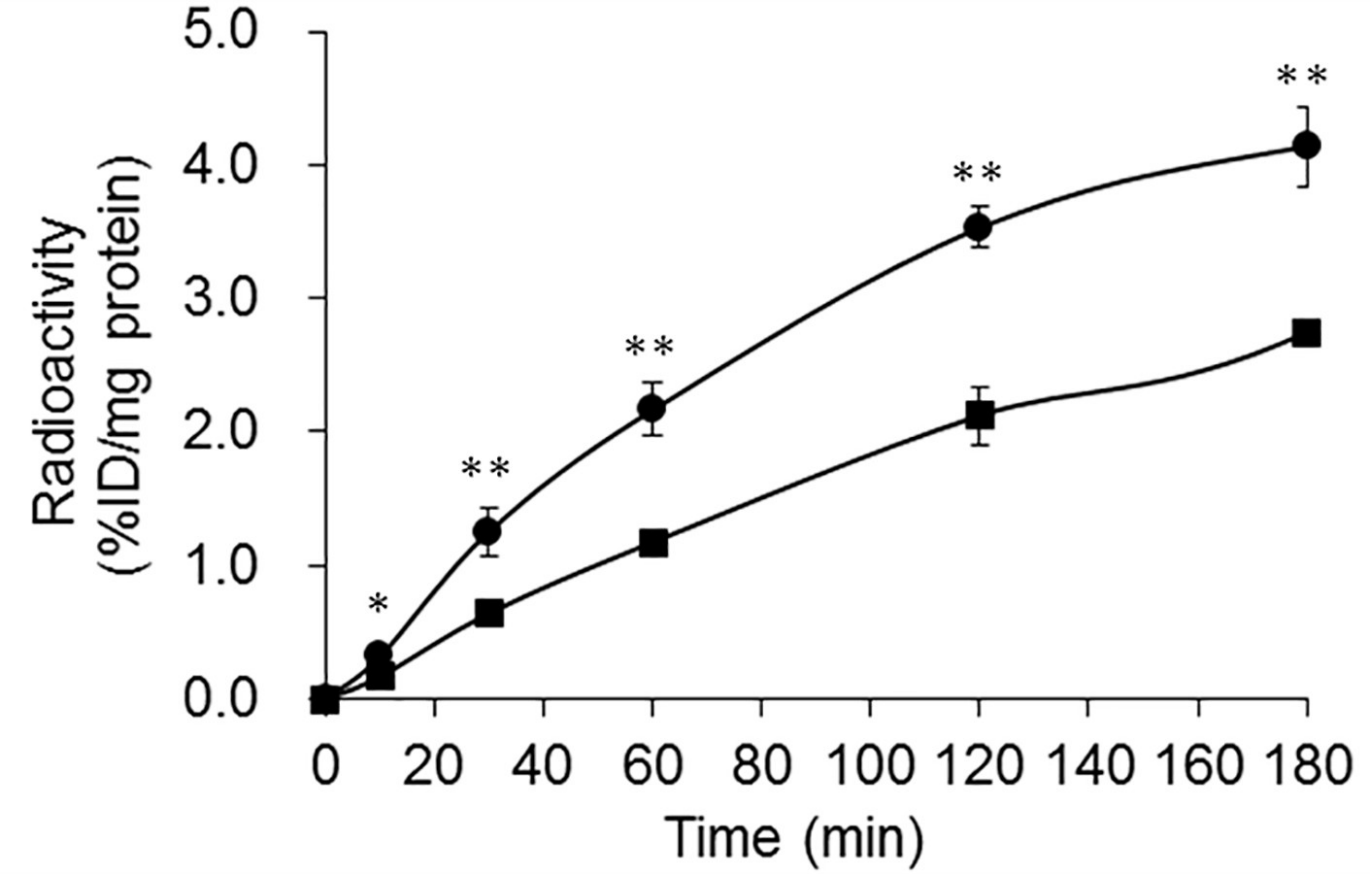
In vitro assays of ^111^In-labeled 3-arm DOTA-α-MSH (■) and 4-arm DOTA-α-MSH (●) in B16-F1 melanoma cells. ^111^In-labeled 4-arm DOTA-α-MSH was taken up early into B16-F1 cells and showed higher accumulation than ^111^In-labeled 3-arm DOTA-α-MSH after 10 min of incubation. **P < 0.01 and *P < 0.05 between ^111^In-labeled 3-arm DOTA-α-MSH and 4-arm DOTA-α-MSH. https://doi.org/10.6084/m9.figshare.7697885.

The accumulation of ^111^In-labeled 3-arm and 4-arm DOTA-α-MSH in B16-F1 cells is shown in Fig 3. ^111^In-labeled 4-arm DOTA-α-MSH was taken up early into the B16-F1 cells and accumulated more than ^111^In-labeled 3-arm DOTA-α-MSH after 10 min of incubation.

### Stability analysis of ^111^In-labeled 3-arm and 4-arm DOTA-α-MSH in mice

R_f_ values of some metabolites were 0.10–0.30. Few metabolites were seen at 10 and 30 min after injection (Table 1). At 60 min after injection, the fraction ratios of ^111^In-labeled 3-arm DOTA-α-MSH were about 86.4%, 84.3%, and 85.7% in the plasma, liver, and kidney of the mice, respectively, whereas the fraction ratios of ^111^In-labeled 4-arm DOTA-α-MSH were about 92.1%, 91.3%, and 93.3%, respectively. At 120 min after injection, the fraction ratios of ^111^In-labeled 3-arm DOTA-α-MSH were about 79.2%, 71.6%, and 78.5% in the plasma, liver, and kidney of the mice, respectively, whereas the fraction ratios of ^111^In-labeled 4-arm DOTA-α-MSH were about 84.2%, 78.6%, and 84.5%, respectively. At 180 min after injection, the fraction ratios of ^111^In-labeled 3-arm DOTA-α-MSH were about 73.5%, 62.9%, and 74.3% in the plasma, liver, and kidney of the mice, respectively, whereas the fraction ratios of ^111^In-labeled 4-arm DOTA-α-MSH were about 80.8%, 76.2%, and 80.5%, respectively.

**Table 1:** Stability analysis of ^111^In-labeled 3-arm and 4-arm DOTA-α-MSH in plasma, liver, and kidney of mice by ex vivo.

	Time (min)	10	30	60	120	180
^111^In labeled 3-arm DOTA-α-MSH	Plasma (%)	96.1 ± 2.0	95.1 ± 1.8	86.4 ± 2.5	79.2 ± 2.2	73.5 ± 2.4
	Liver (%)	95.7 ± 2.1	95.5 ± 1.9	84.3 ± 2.2	71.6 ± 4.5	62.9 ± 6.7
	Kidney (%)	95.8 ± 2.0	95.9 ± 1.7	85.7 ± 2.8	78.5 ± 2.7	74.3 ± 4.1
^111^In labeled 4-arm DOTA-α-MSH	Plasma (%)	96.0 ± 2.5	95.8 ± 2.6	92.1 ± 2.1	84.2 ± 2.2	80.8 ± 2.3
	Liver (%)	95.1 ± 2.9	94.9 ± 2.1	91.3 ± 3.2	78.6 ± 4.9	76.2 ± 4.1
	Kidney (%)	96.1 ± 2.0	95.8 ± 1.9	93.3 ± 3.5	84.5 ± 3.3	80.5 ± 2.8

All data are the mean ± standard deviation from triplicate measurements using three mice per time point

https://doi.org/10.6084/m9.figshare.7697815

### Biological distribution and whole-body SPECT imaging of ^111^In-labeled 3-arm and 4-arm DOTA-α-MSH in B16-F1-bearing mice

**Fig 4:**
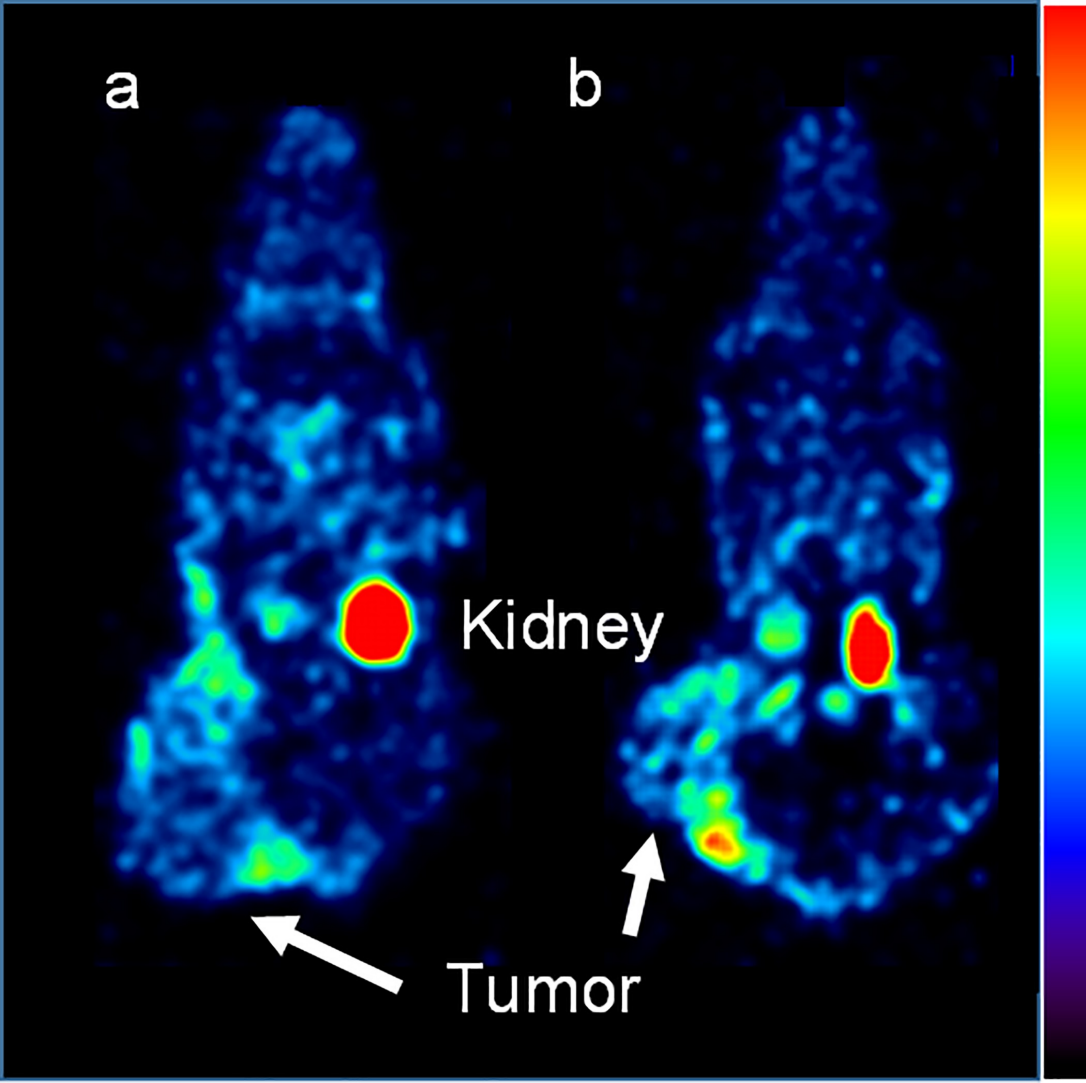
Whole-body SPECT images of B16-F1-bearing mice under 2.0% isoflurane anesthesia injected with 37 MBq ^111^In-labeled 3-arm DOTA-α-MSH (a) or ^111^In-labeled 4-arm DOTA-α-MSH (b) at 170–180 min after injection. The highest accumulation was seen in the tumors and kidney for both radiotracers. We observed that the tumor accumulation of ^111^In-labeled 4-arm DOTA-α-MSH was higher than that of ^111^In-labeled 3-arm DOTA-α-MSH. https://doi.org/10.6084/m9.figshare.7697897.

The biological distributions of ^111^In-labeled 3-arm DOTA-α-MSH (Table 2) and 4-arm DOTA-α-MSH (Table 3) were obtained in B16-F1 melanoma-bearing mice. Radioactivity in the liver, kidney, and blood rapidly increased immediately after injection and then gradually decreased. In the lung and melanoma, the radioactivity reached a peak at 30–60 min after injection. Very little radioactivity was present in other organs. In kidney, ^111^In-labeled 4-arm DOTA-α-MSH provided significantly lower average accumulation (1.38-fold) than ^111^In-labeled 3-arm DOTA-α-MSH at all acquisition time points, whereas ^111^In-labeled 4-arm DOTA-α-MSH provided significantly higher average accumulation (1.32-fold) than ^111^In-labeled 3-arm DOTA-α-MSH in B16-F1 melanoma. Whole-body SPECT images of B16-F1-bearing mice were obtained for ^111^In-labeled 3-arm (a) and 4-arm DOTA-α-MSH (b) at 170–180 min after injection (Fig 4). The main accumulation was found in kidney and B16-F1 tumors in images. In particular, accumulation of ^111^In-labeled 4-arm DOTA-α-MSH was higher than that of ^111^In-labeled 3-arm DOTA-α-MSH in B16-F1 melanoma. In other organs, little accumulation of ^111^In-labeled 3-arm DOTA-α-MSH and ^111^In-labeled 4-arm DOTA-α-MSH was observed.

**Table 2:** Biological distribution of ^111^In-labeled 3-arm DOTA-α-MSH.

Organ (%ID/g or %ID/organ)	10 min			30 min			60 min			120 min			180 min		
Brain	0.04	±	0.01	0.05	±	0.02	0.05	±	0.01	0.03	±	0.00	0.02	±	0.00
Lung	0.51	±	0.15	0.66	±	0.24	0.31	±	0.09	0.27	±	0.09	0.10	±	0.04
Heart	0.74	±	0.15	0.66	±	0.13	0.46	±	0.15	0.14	±	0.06	0.06	±	0.02
Stomach*	0.06	±	0.03	0.10	±	0.03	0.12	±	0.03	0.11	±	0.05	0.12	±	0.03
Liver	2.30	±	1.04	1.15	±	0.92	1.12	±	0.86	1.23	±	0.47	0.72	±	0.15
Small intestine*	0.04	±	0.03	0.08	±	0.02	0.09	±	0.03	0.12	±	0.03	0.10	±	0.03
Large intestine*	0.03	±	0.01	0.08	±	0.02	0.09	±	0.03	0.05	±	0.03	0.04	±	0.03
Kidney	23.55	±	3.63	18.42	±	5.57	11.87	±	5.40	8.42	±	4.27	7.07	±	3.02
Blood	3.11	±	0.84	1.91	±	0.45	0.89	±	0.22	0.21	±	0.06	0.05	±	0.02
Muscle	0.21	±	0.07	0.20	±	0.08	0.15	±	0.08	0.09	±	0.04	0.05	±	0.02
Melanoma	1.71	±	0.64	1.81	±	0.45	2.49	±	0.62	3.36	±	0.90	3.51	±	0.78

%ID/g indicates percent injected dose per gram of tissue.

*%ID/organ was calculated from %ID per organ. Values are the average ± standard deviation.

Values are the mean ± standard deviation obtained from three mice per time point.

https://doi.org/10.6084/m9.figshare.7698227

**Table 3:** Biological distribution of ^111^In-labeled 4-arm DOTA-α-MSH.

Organ (%ID/g or %ID/organ)	10 min			30 min			60 min			120 min			180 min		
Brain	0.04	±	0.01	0.03	±	0.01	0.03	±	0.01	0.02	±	0.00	0.01	±	0.00
Lung	0.42	±	0.12	0.52	±	0.14	0.22	±	0.08	0.13	±	0.04	0.06	±	0.01
Heart	0.55	±	0.11	0.46	±	0.10	0.38	±	0.08	0.14	±	0.03	0.05	±	0.01
Stomach*	0.09	±	0.02	0.09	±	0.03	0.08	±	0.02	0.05	±	0.01	0.05	±	0.01
Liver	2.52	±	0.73	1.45	±	0.74	1.18	±	0.69	0.90	±	0.33	0.61	±	0.12
Small intestine*	0.05	±	0.01	0.04	±	0.01	0.03	±	0.01	0.02	±	0.00	0.02	±	0.00
Large intestine*	0.03	±	0.01	0.03	±	0.02	0.04	±	0.01	0.03	±	0.01	0.03	±	0.01
Kidney	17.14	±	4.12	13.33	±	3.87	8.12	±	3.11	6.31	±	2.11	5.19	±	1.82
Blood	4.03	±	0.93	2.02	±	0.54	1.01	±	0.28	0.11	±	0.04	0.03	±	0.00
Muscle	0.10	±	0.03	0.12	±	0.05	0.08	±	0.02	0.04	±	0.00	0.03	±	0.00
Melanoma	2.22	±	0.65	2.25	±	0.63	3.34	±	0.93	4.52	±	1.01	4.83	±	1.03

%ID/g indicates percent injected dose per gram of tissue.

*%ID/organ was calculated from %ID per organ. Values are the average ± standard deviation.

Values are the mean ± standard deviation obtained from three mice per time point.

https://doi.org/10.6084/m9.figshare.7698230

Table 4 summarizes melanoma-to-kidney and melanoma-to-muscle ratios. The average melanoma-to-kidney ratios for ^111^In-labeled 3-arm DOTA-α-MSH were 0.07, 0.10, 0.21, 0.40, and 0.50 at 10, 30, 60, 120, and 180 min after injection, respectively. For ^111^In-labeled 4-arm DOTA-α-MSH, the ratios were 0.13, 0.17, 0.41, 0.72, and 0.93, respectively. The average melanoma-to-blood ratios for ^111^In-labeled 3-arm DOTA-α-MSH were 0.55, 0.95, 2.80, 16.00, and 65.00 at 10, 30, 60, 120, and 180 min after injection. For ^111^In-labeled 4-arm DOTA-α-MSH, the ratios were 0.55, 1.11, 3.31, 41.09, and 155.81, respectively. The average melanoma-to-muscle ratios for ^111^In-labeled 3-arm DOTA-α-MSH were 8.14, 9.05, 16.60, 37.33, and 66.23 at 10, 30, 60, 120, and 180 min after injection. For ^111^In-labeled 4-arm DOTA-α-MSH, the ratios were 22.20, 18.75, 41.75, 113.00, and 155.81, respectively.

**Table 4:** Melanoma-to-organ ratios of ^111^In-labeled 3-arm and 4-arm DOTA-α-MSH in B16-F1-bearing mice.

Time (min)	Melanoma-to-kidney		Melanoma-to-blood		Melanoma-to-muscle	
	3-arm DOTA-α-MSH	4-arm DOTA-α-MSH	3-arm DOTA-α-MSH	4-arm DOTA-α-MSH	3-arm DOTA-α-MSH	4-arm DOTA-α-MSH
10	0.07 ± 0.03	0.13 ± 0.07*	0.55 ± 0.13	0.55 ± 0.14	8.14 ± 1.44	22.20 ± 3.35**
30	0.10 ± 0.04	0.17 ± 0.11**	0.95 ± 0.30	1.11 ± 0.28*	9.05 ± 1.38	18.75 ± 4.45**
60	0.21 ± 0.04	0.41 ± 0.15**	2.80 ± 0.58	3.31 ± 0.35**	16.60 ± 3.28	41.75 ± 6.38**
120	0.40 ± 0.09	0.72 ± 0.18**	16.00 ± 3.21	41.09 ± 4.78**	37.33 ± 4.23	113.00 ± 7.33**
180	0.50 ± 0.10	0.93 ± 0.15**	65.00 ± 4.64	155.81 ± 12.93**	66.23 ± 4.21	155.81 ± 8.63**

All data were mean ± standard deviation measured in four mice.

**P < 0.01 and

*P < 0.05 between ^111^In labeled 3-arm DOTA-α-MSH and 4-arm DOTA-α-MSH.

https://doi.org/10.6084/m9.figshare.7698239

## Discussion

In this study, the 4-arm DOTA construct was newly applied for conjugation to α-MSH to evaluate specific activity, radiolabeling efficiency, MC1-R affinity, stability, and tumor accumulation in melanoma imaging. Although specific activity and radiolabeling efficiency of ^111^In-labeled 4-arm DOTA-α-MSH were not much different from those of 3-arm DOTA-α-MSH, which is the generally used DOTA construct, specific activity was quite low compared to labeling with other MC1-R analogues [14,20]. Because the partition coefficients of ^111^In-labeled 3-arm and 4-arm DOTA-α-MSH were negative, these radiotracers are water soluble and are usually renally excreted. We observed high renal accumulation and excretion of ^111^In-labeled 3-arm and 4-arm DOTA-α-MSH (Fig 4). However, the lipophilicity of ^111^In-labeled 4-arm DOTA-α-MSH was slightly closer to zero than that of ^111^In-labeled 3-arm DOTA-α-MSH according to the partition coefficients. We estimated that the lipophilicity resulted in a slightly different distribution between ^111^In-labeled 3-arm DOTA-α-MSH and ^111^In-labeled 4-arm DOTA-α-MSH (Tables 2 and 3).

In the inhibition study (Fig 2), because accumulation of both ^111^In-labeled 3-arm and 4-arm DOTA-α-MSH was significantly inhibited by the substrate, α-MSH, in B16-F1 cells with high MC1-R expression, and not inhibited in 4T1 cells with low MC1-R expression, both radiotracers bound to MC1-R. Although the inhibition rate of ^111^In-labeled 4-arm DOTA-α-MSH was lower than that of ^111^In-labeled 3-arm DOTA-α-MSH at 180 min after incubation, the amount of inhibition of ^111^In-labeled 4-arm DOTA-α-MSH (mean 3.28) was higher than that of ^111^In-labeled 3-arm DOTA-α-MSH (mean 2.67) according to the calculation from Figs 2 and 3. Therefore, nonspecific accumulation of ^111^In-labeled 4-arm DOTA-α-MSH (mean 0.92) was higher than that of ^111^In-labeled 3-arm DOTA-α-MSH (mean 0.23). This moderate nonspecific accumulation is estimated to be caused by lipophilicity of ^111^In-labeled 4-arm DOTA-α-MSH. In Fig 3, the time-activity curve of ^111^In-labeled 4-arm DOTA-α-MSH was significantly higher than that of ^111^In-labeled 3-arm DOTA-α-MSH in B16-F1 melanoma cells. In the in vitro study, the high accumulation of ^111^In-labeled 4-arm DOTA-α-MSH may be caused by the effect of lipophilicity and high affinity for MC1-R in B16-F1 melanoma cells.

Moderate stability of ^111^In-labeled 3-arm and 4-arm DOTA-α-MSH was found at all time points (Table 1). The stability of ^111^In-labeled 4-arm DOTA-α-MSH was higher than that of ^111^In-labeled 3-arm DOTA-α-MSH. In addition, these stabilities were relatively higher than another peptide radiotracer conjugated to 3-arm DOTA [21]. One reason may be that eight-coordinate complexes of ^111^In-labeled 4-arm DOTA conjugation are generally more stable than seven-coordinate complexes of ^111^In-labeled 3-arm DOTA conjugation [22, 23].

In the biological distribution of ^111^In-labeled 3-arm (Table 2) and 4-arm DOTA-α-MSH (Table 3) and whole-body SPECT imaging (Fig 4), accumulation of ^111^In-labeled 4-arm DOTA-α-MSH was significantly higher than that of ^111^In-labeled 3-arm DOTA-α-MSH in B16-F1 melanoma. This also supports the similar results from the in vitro study in Fig 3. ^111^In-labeled 4-arm DOTA-α-MSH showed significantly lower renal accumulation than ^111^In-labeled 3-arm DOTA-α-MSH at all acquisition times. The number of carboxy groups affects electric charges in the whole body and changes the whole-body distribution of peptide radiotracers [24]. Thus, the effect of electric charges may yield lower renal accumulation. For peptide radiotracers, reducing renal accumulation is important. Behr et al. showed that systemic administration of cationic amino acids reduces renal reabsorption and accumulation of peptide radiotracers [25]. However, amino acid infusion may change the whole-body distribution and increase background accumulation in muscle, blood, and kidney etc. On the other hand, ^111^In-labeled 4-arm DOTA-α-MSH showed significantly higher melanoma accumulation than ^111^In-labeled 3-arm DOTA-α-MSH. This may be because of the slightly higher lipophilicity, significantly higher MC1-R affinity, and relatively higher stability of 4-arm DOTA-α-MSH compared to 3-arm DOTA-α-MSH. If renal accumulation is reduced and tumor accumulation is increased using other peptides conjugated to the 4-arm DOTA construct compared with the 3-arm DOTA construct, the 4-arm construct will be a simple and useful DOTA construct for labeling of peptide radiotracers.

The melanoma-to-kidney, melanoma-to-blood, and melanoma-to-muscle ratios (Table 4) of ^111^In-labeled 4-arm DOTA-α-MSH were significantly higher than those ratios of ^111^In-labeled 3-arm DOTA-α-MSH. However, these ratios were relatively lower than those of other ^111^In-labeled DOTA peptides [12] because we selected the native α-MSH peptide hormone to evaluate the chemical properties between the 3-arm DOTA and 4-arm DOTA constructs. We selected native α-MSH as a good peptide to evaluate improvement of chemical properties using the 4-arm DOTA construct because 3-arm DOTA-α-MSH has relatively low yield, MC1-R affinity, and stability compared to other 3-arm DOTA-conjugated peptides [14]. The IC_50_ values of ^111^In-labeled 3-arm and 4-arm DOTA-α-MSH were 4.8 ± 0.5 nM and 5.3 ± 0.4 nM, respectively. These are similar to or better than those of numerous ^111^In-labeled α-MSH analogues (0.9–78.6 nM) [12]. Therefore, utilization of α-MSH for comparison of ^111^In-labeled 3-arm and 4-arm DOTA peptides was considered appropriate. However, when other peptides are applied for ^111^In labeling of the 4-arm DOTA construct, melanoma-to-tissue ratios will improve more than other ^111^In-labeled DOTA peptides with the 3-arm DOTA construct. Thus, 4-arm DOTA peptides will be effective for tumor diagnosis because of their slightly higher lipophilicity, higher MC1-R affinity, and relatively higher stability compared to 3-arm DOTA peptides. https://doi.org/10.6084/m9.figshare.7698239

For internal radiation therapy, we can use 4-arm DOTA constructs with ^225^Ac labeling instead of ^111^In. ^225^Ac-labeled 4-arm DOTA peptides are estimated to form eight-coordinate complexes similar to ^111^In-labeled 4-arm DOTA peptides because the chemical characteristics and properties of ^225^Ac are similar to those of ^111^In [26]. Therefore, ^225^Ac-labeled 4-arm DOTA peptides may provide higher melanoma accumulation, better internal radiation therapy, and relatively lower renal accumulation, similar to ^111^In-labeled 4-arm DOTA peptides.

## Conclusion

^111^In-labeled 4-arm DOTA-α-MSH yields higher tumor accumulation and lower renal accumulation than ^111^In-labeled 3-arm DOTA-α-MSH because the chemical properties of the 4-arm DOTA construct include slightly higher lipophilicity, significantly higher MC1-R affinity, and relatively higher stability. Therefore, 4-arm DOTA constructs provide better chemical properties for peptide radiotracers than 3-arm DOTA constructs.
